# FFAR from the Gut Microbiome Crowd: SCFA Receptors in T1D Pathology

**DOI:** 10.3390/metabo11050302

**Published:** 2021-05-11

**Authors:** Medha Priyadarshini, Kristen Lednovich, Kai Xu, Sophie Gough, Barton Wicksteed, Brian T. Layden

**Affiliations:** 1 Department of Medicine, Division of Endocrinology, Diabetes, and Metabolism, University of Illinois at Chicago, Chicago, IL 60612, USA; mpriya2@uic.edu (M.P.); kledno2@uic.edu (K.L.); kxu12@uic.edu (K.X.); sgough2@uic.edu (S.G.); bartonw@uic.edu (B.W.); 2 Jesse Brown Veterans Affair Medical Center, Chicago, IL 60612, USA

**Keywords:** free fatty acid receptor (FFA) 2, FFA3, gut microbiome, incretin, insulin secretion, short-chain fatty acids, type 1 diabetes

## Abstract

The gut microbiome has emerged as a novel determinant of type 1 diabetes (T1D), but the underlying mechanisms are unknown. In this context, major gut microbial metabolites, short-chain fatty acids (SCFAs), are considered to be an important link between the host and gut microbiome. We, along with other laboratories, have explored how SCFAs and their cognate receptors affect various metabolic conditions, including obesity, type 2 diabetes, and metabolic syndrome. Though gut microbiome and SCFA-level changes have been reported in T1D and in mouse models of the disease, the role of SCFA receptors in T1D remains under explored. In this review article, we will highlight the existing and possible roles of these receptors in T1D pathology. We conclude with a discussion of SCFA receptors as therapeutic targets for T1D, exploring an exciting new potential for novel treatments of glucometabolic disorders.

## 1. Introduction

T1D is an organ-specific autoimmune disease characterized by the destruction of the β cells. While the etiology of T1D is not fully understood, the prevailing paradigm hypothesizes that an individual’s genetic background plays a central role in the risk of disease development, and more than 50 T1D-associated genes have been identified through extensive genetic studies [1]. However, genetic predisposition alone cannot explain recent rises in the rates of T1D worldwide. Environmental factors, including mode of delivery during birth [2], breast versus formula feeding [2], use of antibiotics in early life [3,4], toxicant exposure [5], etc., have lately emerged as factors potentially affecting T1D onset and progression. Of note, most of these factors converge upon the gut microbiome (GM), making this a novel critical factor, the modulation of which can accelerate or offset T1D progression [6,7].

Comprising over 10 trillion cells and outnumbering host cells approximately 10:1, the GM refers to the totality of microorganisms, including bacteria, fungi, archaea, and viruses, that inhabit the intestinal tract [8]. The vast majority of gut microbes play either commensal or mutualistic roles, including nutrient absorption and digestion, the regulation of endocrine functions and signaling to the brain, eliminating toxins, and producing crucial metabolites for the body. Importantly, the GM is the major generator of short-chain fatty acids (SCFAs) through the intestinal fermentation of dietary complex carbohydrates that cannot be metabolized by the host. SCFAs, along with their role as a nutrient, have an expansive repertoire of functional roles, including the regulation of glucose, lipid, and energy metabolism, modulation of gene expression, cell proliferation, and inflammation, and have localized effects on intestinal function [9]. Additionally, SCFAs act as major mediators of crosstalk between the GM and human host, as fluctuating levels of SCFAs are influenced by changes in gut microbial composition and are largely impacted by factors such as diet and physiological state. Bridging the environment–gut microbiome–host axis, it is not surprising that SCFAs have emerged as major factors in the development of metabolic disorders [10]. Their role in T1D has been recognized recently and is discussed elsewhere [11].

The numerous actions of SCFAs are carried out, in part, through interaction with their membrane receptors, which act both as sensors and mediators of their effects [12]. Two of the important SCFA receptors are free fatty acid receptors (FFAR), FFA2 and FFA3, which belong to the G-protein-coupled receptor family. With widespread tissue expression, the roles of FFA2 and FFA3 as major regulators of metabolism and immunity, as well as in the mediation of microbiome–host crosstalk, are emerging [12]. As discussed in subsequent sections, this is accomplished through a variety of mechanisms, including the modulation of insulin secretion in the pancreas, incretin secretion in the intestine, and the modulation of inflammatory responses. Furthermore, there is a growing body of data supporting the involvement of SCFA receptors in the pathogenesis of T1D [13]. In this review, we also explore their involvement in the pathogenesis of T1D by summarizing the current body of data derived from experimental mouse models and identify areas of interest for future research in this direction.

## 2. Gut Microbiome in T1D

Our understanding of the role of GM in T1D is based on rodent and human studies (summarized in [7,14]). In general, the intestinal microbiota of T1D and healthy subjects are distinctly different in mouse and human studies, with the former showing reduced microbial diversity and a reduced presence of butyrate producers along with proinflammatory dysbiosis [7,15]. For example, in Non-obese Diabetic (NOD) mice (susceptible to developing diabetes in an autoimmune fashion similar to humans) and Non-obese Diabetes Resistant (NOR) mice, the NOD microbiota contained more pathobionts compared to beneficial bacteria present in the NOR. The ileal NOD microbiota was reduced in segmented filamentous bacteria (SFB) and *Lactobacillus*spp., while the ileal NOR microbiota were reduced in *Anaeroplasma*spp. and *Desulfovibrio*spp. The colonic NOD microbiota was reduced in members of the Alphaproteobacteria class, *Ruminococcus gnavus*, and absent in *Bacteroides acidfaciens* when compared to NOR mice. The colonic NOR microbiota were absent in *Prevotella*spp. but detected in NOD mice. Importantly, SFB and *Lactobacillus* are associated with protection against the development of autoimmune diabetes [16,17], while expansion of strains such as *Prevotella* correlates with detrimental changes in the gut mucosal immune system [18]. The transfer of NOD gut microbes promoted pancreatic inflammation in NOR mice [19]. Blocking the interaction between GM and NOD host by genetic knockout of MYD88, an adapter protein responsible for activating receptors for gut microbial signals and lodging appropriate innate immune response, protected against T1D development. Interestingly, this protective effect was lost in absence of the gut microbiome and could be gained back through recolonization with NOD MYD88 knockout gut microbiota [20]. These results suggested that while the interaction of the GM with the innate immune system is a predisposing factor for T1D [20], it is the balance in gut microbial features affecting tolerance versus T1D development that affects the disease pathogenesis.

Gut microbial changes occurring in T1D have also been analyzed in human cohorts. Two studies utilizing the Environmental Determinants of Diabetes in the Young (TEDDY) cohort have yielded a functional profile of the developing gut metagenome, identifying the relationship between the microbiome and islet immunity and T1D, as well as other major childhood events [21,22]. Similar to observations in NOD mice, control children had higher beneficial bacteria such as *Bifidobacterium* and *Lactobacillus* [21,22], while in children with islet autoimmunity and T1D, there was a high prevalence of *Erythisopelotrichaecae* and fewer *Lactococcus*, *Streptococcus*, and *Akkermansia* [22]. Additionally, the GM of control children had more genes associated with fermentation and biosynthesis of SCFAs [21]. An earlier study comparing children prior to T1D onset (defined as presence of at least two β-cell-specific autoantibodies) with autoantibody-negative children also demonstrated that individuals with a greater number of autoantibodies to β cell antigens had a lower abundance of lactate and butyrate-producing gut bacteria [23].

Despite some key differences in rodent and human T1D forms [24], a pattern of GM modulation in the disease has emerged [7]. Crucial in this pattern are the observations that (a) deviation from optimal microbial homeostasis may lead to loss of self-tolerance and rise in proinflammatory signals [19,21,22,25], and (b) functional net effects of these deviations depend on co-occurring microbial communities and have often been manifested as changes in levels of plasma and fecal SCFAs [19,21,26,27]. In the animal form of T1D, the restoration of optimal GM community (either nutritionally or through fecal microbial transfer, FMT) alleviates and/or delays features of severe forms of the disease [15,28,29]. Similarly, GM restoration in human T1D has shown the benefits of an optimal GM community [14,30]. However, the interrelationship between GM changes and T1D, though apparent, has not been proven causal.

## 3. SCFAs, FFA2, and FFA3

SCFAs, mainly acetate, butyrate, and propionate, are gut microbiota fermentation byproducts of indigestible fiber. The majority of SCFAs are readily absorbed and utilized by the colonocytes as an energy source. Remaining SCFAs are drained into the hepatic and portal venous systems before emerging into the systemic circulation [31]. SCFAs affect host physiology in numerous ways, acting both as metabolic substrates and signaling molecules. Distinct GM and SCFA profiles in T1D versus controls provide compelling evidence for the roles of SCFA receptors in disease pathology. For a better understanding of this role, a review of known functions of FFA2 and FFA3 centering on the endocrine pancreas, immune cells, and gut is presented. Discussion of the roles of these receptors in other tissues has been covered elsewhere [32,33,34].

### 3.1. SCFAs as Signaling Molecules

SCFAs act as extracellular signaling molecules by binding to their cognate G-protein-coupled receptors (GPCRs), FFA2 and FFA3, which can bind all the three SCFAs but with discrete efficacies (Table 1) and G-protein coupling profiles. Due to their coexpression in tissues, shared endogenous ligands, and lack of selective synthetic ligands, defining their physiological roles has been challenging. However, recent studies using novel rodent models have provided an increased appreciation of the roles of these receptors in various metabolic and immune states [12].

### 3.2. FFA2 and FFA3 Regulate β Cell Physiology

Both FFA2 and FFA3 are expressed in islets, predominantly in the β cells in both rodents and humans [34]. Pioneering work from the Layden Laboratory along with others has established the role of these receptors in the regulation of β cell function and mass [38,39,40,41,42,43,44]. Most of these effects are based upon distinct G-protein coupling preferences of FFA2 and FFA3. Upon SCFA binding, FFA2 can couple with either Gα_q/11_ and Gα_i/o_, thus exerting stimulatory or inhibitory effects on cellular function, respectively. FFA3, on the other hand, couples almost exclusively with Gα_i/o_, with an inhibitory tone in its signaling [36]. Accordingly, in the islet, these receptors have opposing effects on insulin secretion: in both human and mouse islets, FFA3 inhibits insulin secretion in a Gα_i/o_-dependent manner [38,42,45], whereas FFA2 activation may increase [39,44,46,47] or decrease insulin secretion [45], depending upon whether it couples to Gα_q/11_ or Gα_i/o_. Variance observed in FFA2 activity suggests that under any given condition, the effect of FFA2 activation on insulin secretion depends upon its preferred G-protein coupling [39,44]. This calls for the development of G-protein-biased ligands for FFA2. In fact, orthosteric FFA2 agonists SCA14, SCA15, and ZINC03832747 mediate the Gα_q/11_-dependent increase in mouse islets or β cell insulin secretion in contrast to the allosteric agonists CMTB and CPTB that decrease insulin secretion via Gα_i/o_ [39,48].

Mediation of β cell function by these receptors projects a similar profile in vivo. Whole-body deletion of FFA3 improves insulin secretion and glucose tolerance both under high fat diet induced metabolic stress [42,43,45] and a regular diet [42,43]. Correspondingly, β-cell-specific FFA3 overexpression deteriorates glucose responsiveness in mice [42]. These effects appear to be a β cell secretory phenotype, as no changes in insulin sensitivity have been observed [42,43,45]. Additionally, gene expression analysis of islets from FFA3 knockout mice [39] or β-cell-specific FFA3 overexpression mice [42] revealed complementary changes (i.e., downregulation in knockout and upregulation in overexpression model) in genes related to inflammation and immune response (such as IL1β, IL1α, CD80), besides changes in genes of calcium response and glucose utilization pathways.

Evaluation of the in vivo roles of FFA2, similar to the ex vivo data, has yielded conflicting results. Mice globally lacking FFA2 exhibited fasting hyperglycemia, reduced insulin secretion, and glucose intolerance under dietary metabolic stress [39,44]. In contrast, another study has reported a phenotype of improved glucose tolerance and enhanced insulin secretion in FFA2 knockout mice [45]. Additionally, in this same study, FFA2 and FFA3 double knockout or FFA3 knockout in combination with β-cell-specific FFA2 knockout improved glucose tolerance and insulin secretion under metabolic stress. These conflicting data may arise from differences in the G-protein coupling of activated FFA2, roles of FFA2 in other metabolically active tissues, impact of gut microbiome, and/or duration of metabolic stress, besides receptor-independent effects of SCFAs [49,50,51].

SCFA receptors, specifically FFA2, also modulate β cell mass [40,41,44]. FFA2 is required for the prenatal establishment of β cell mass, as FFA2 knockout mouse neonates and 21-day-old weanlings exhibit impaired β cell mass at birth and throughout adulthood [41]. Under conditions of dietary metabolic stress [44] and pregnancy [40], when β cells are compensating for insulin resistance, this deficiency in β cell mass is magnified. FFA2 activation, as a matter of fact, increases β cell proliferation [41,44], enhances the expression of genes involved in β cell differentiation [44], and reduces cytokine- and palmitate-induced β cell apoptosis [46,47]. FFA3, on the other hand, as a Gα_i/o_-coupling receptor, may restrict β cell mass [52]. However, FFA3 knockout mice islets have been reported to be smaller with reduced proliferation and number of β cells [42], an effect not seen in a later study [43]. Similarly, β-cell-specific FFA3 overexpression in mice shows compensatory increased β cell proliferation and area [42]. Collectively, these data highlight the role of SCFA receptors FFA2 and FFA3 in modulating β cell function and mass. Importantly, defects in these two features are fundamental to the pathology of T1D.

### 3.3. FFA2 and FFA3 Modulate Incretin Secretion

In addition to their role within pancreatic islets, SCFA and their receptors are suggested to participate in the secretion of incretin hormones. In the upper intestine, SCFA concentrations range from 0.1 to 1 mM and are largely produced by oral microbiota [53]. By contrast, luminal SCFAs in the colon can reach levels of up to 100 mM due to the fermentation of dietary fibers via the gut microbiota. Within the intestine, SCFA receptors FFA2 and FFA3 are thought to act as sensors of these metabolites, and many important actions are carried out through this signaling.

Secreted by enteroendocrine cells (EECs) embedded within the intestinal epithelium, incretin hormones are peptide hormones that stimulate the release of insulin in response to nutrient intake, thereby lowering the level of circulating blood glucose [54]. Additionally, incretin hormones facilitate numerous postprandial metabolic functions, including lowering food intake, gastric emptying, and increasing cardiac output [55]. There are two primary incretin hormones: glucagon-like peptide-1 (GLP-1) and gastric inhibitory polypeptide (GIP). While both hormones carry out their various functions through the binding of their specific receptors (GLP-1R and GIPR, respectively) on the surface of various tissues, they contribute to the regulation of glucose metabolism in distinct mechanisms. Although both stimulate insulin release through Gα/cAMP at β cells, in islet α cells, GLP-1 suppresses glucagon, while GIP increases it [56]. Both hormones also protect β cell mass by inhibiting apoptosis. GLP-1 and GIP play important roles in the control of glucose levels after a meal via the physiological response known as the “incretin effect.” This occurs when higher levels of glucose-stimulated insulin secretion are observed when glucose is administered orally rather than intravenously, an effect that is lost in type 2 diabetes but preserved in T1D [57].

FFA2 and FFA3 are broadly expressed within EECs throughout the gastrointestinal tract. EECs are divided into subtypes based on the peptide hormone they express and secrete [58]. Using immunohistochemical analysis and a *Ffar2*-red fluorescent protein (RFP) reporter mouse, FFA2 has been found to colocalize with peptide YY (PYY)/GLP-1 containing L cells in rodents and humans [59,60,61,62]. Using in situ hybridization and a *Ffar3*-RFP reporter mouse, FFA3 expression has been confirmed in several types of intestinal cells, including PYY-positive cells [59,63,64]. However, due to the concentrated expression of FFA3 in enteric ganglia and sympathetic ganglia, it is uncertain if its effects on EEC function arise from its expression in enterocytes or are secondary to its mediation of enteric neuronal function [59,65,66]. Further, transcriptomic analysis has found high coexpression of both receptors with *gip*, indicating their possible involvement in mediating GIP secretion [67].

Several studies have shown that stimulation of FFA2 by SCFA results in an increased secretion of GLP-1 from EECs in the intestine. Primary intestine cells harvested from global FFA2 knockout mice showed reduced GLP-1 release in vitro, and another study by the same group found that propionate was able to stimulate GLP-1 release in vivo only in the wild-type mice [68,69]. Besides GLP-1, studies have also documented the role of FFA2 in mediating GIP [44] and PYY secretion, with the latter in both humans and mice [44,70,71]. Controversy exists for this role of FFA2, however, with some studies reporting no difference in basal- and glucose-stimulated GLP-1 levels in FFA2 knockout mice compared to control mice [40,45].

For FFA3, while there is a paucity of data regarding its role in gut hormone secretion, FFA3 knockout mice have reduced GLP-1 and PYY secretion, and primary colonic cultures derived from these mice display impaired secretory response upon SCFA stimulation [63,69]. Predictably, FFA3-specific agonist enhances GLP-1 release from primary colonic cultures [59]. FFA3 is also implicated in the inhibition of GIP secretion, an effect more likely with the predominant Gα_i/o_ coupling of the receptor [72].

More research is needed to ascertain the respective roles of FFA2 and FFA3 in the regulation of incretin hormones in the intestine. This includes the development of potent selective ligands and tissue-specific knockout mouse models. Some progress has already been made in this direction. Selective and potent human FFA2 inverse agonists have been developed and shown to stimulate GLP-1 secretion in the human EEC line, NCI-H716 [73]. Through a chemogenetic knock-in strategy, mice with designer receptors exclusively activated by the designer drugs (DREADD) variant of human *FFAR2* replacing the mouse *Ffar2* locus have been generated. DREADD activation in these mice has been shown to augment GLP-1 secretion in colonic crypt cultures and in vivo [74].

### 3.4. SCFAs, FFA2, and FFA3 Educate the Gut Immune Cells and Regulate Inflammation

SCFAs can regulate immune cell function via two major processes, either through their cognate GPCRs such as FFA2 and FFA3 or by modulating histone deacetylase (HDAC) activity [75]. Here, we emphasize the first role. With the highest SCFA concentration, a rich and diverse population of immune cells with the majority expressing FFA2 and few, such as dendritic cells expressing FFA3, the gut is an important site where SCFAs can impact the immune cells through these receptors.

FFA2 has been reported to affect neutrophil chemotaxis in gut inflammation models, where a deficiency in FFA2 increases neutrophil infiltration to sites of inflammation [76,77]. Accordingly, engagement of FFA2 with acetate mitigates such a response [76,77]. Similar FFA2-dependent neutrophil-driven responses are seen in pulmonary and joint inflammation models [77]. Neutrophil FFA2 engagement by SCFAs in the presence of allosteric modulators can also activate NADPH oxidase and enhance the production of reactive oxygen species, which is deemed necessary for phagocytic activity [77,78]. FFA2-derived neutrophil responses are required for the regulation of inflammatory responses. As recently shown, FFA2 activity promotes coordination between neutrophils and colonic group 3 innate lymphoid cells (ILC3). In neutrophils, inflammasome activation helps in pathogen clearance with the concomitant enhancement of IL1β production, where IL1β leads to IL22 production from ILC3, driving gut repair mechanisms [79]. FFA2 can also promote ILC3 expansion and function independent of neutrophils [80]. Immunomodulation by neutrophil FFA2, thus, appears to strike a balance between pro- and anti-inflammatory effects, potentially in a disease-centric manner.

SCFAs through their HDAC inhibitory activity are considered to be the main players in maintaining the regulatory T cell (Treg) pools [75]. These effects are, in part, mediated through FFA2. It is suggested that FFA2 exerts immune suppression by regulating the number, function, and differentiation of Tregs [81,82]. FFA2 also modulates gut homeostasis by modifying immunoglobulin A (IgA) production [83] and through direct effects on inflammasome activation in intestinal epithelial cells [84].

The role of FFA3 in immune regulation is less explored, likely due to its limited expression in immune cells. It has been suggested to be involved in the resolution of lung inflammation through effects on macrophage and dendritic cell populations [85] and in promoting thymic Treg differentiation in mouse offspring [86]. The function and expansion of CD8^+^ T cells can also be regulated by FFA3, and this has been suggested to aid the resolution of influenza infection [87]. Both FFA3 and FFA2 have been suggested to enhance T cell memory [88], with the engagement of both receptors by butyrate appearing to mediate this effect. However, as the mouse isoform of FFA2 shows a low affinity for butyrate [31], the use of synthetic ligands is required to further delineate the role of the two receptors in this process.

### 3.5. SCFAs, FFA2, and FFA3, and Gut Microbiome: It Takes Three to Tango

Obliterating the GM in mice wipes off some of the physiological effects discussed above. For instance, in germ-free (GF) mice, antigen-activated T cells fail to transition to memory cells [88]. As the GM does not directly interact with the host cells except at the gut mucosal surfaces, these effects are likely indirect, being mediated via GM-derived factors such as SCFAs. The GM-derived SCFAs acting through their receptors FFA2 and FFA3 project the link, GM→SCFAs→FFA2 and FFA3.

Highlighting this relationship, whole-body FFA2 and FFA3 knockout mice have different gut microbiota profiles as compared to wild-type mice [44,63,82,84,89]. As expected, differences in fecal SCFA profiles accompany these differences in GM profiles due to adaptation to receptor deficiency. More direct evidence for roles of FFA2 and FFA3 in the GM→SCFAs→Receptor link is provided by immune function and GM studies. Both GF and FFA2 knockout mice exhibit a dysregulated immune response to induced colitis, gout, and arthritis [77,90]. While this response is mitigated by acetate supplemented drinking water in GF mice [77], FFA2 knockout mice remain refractory to acetate treatment [81].

Likewise, high-fiber diets that tend to increase GM function and SCFA levels in wild-type mice fail to promote gut homeostasis, alleviate food allergy, and prevent respiratory viral infection in FFA2 knockout mice [82,84,91]. Similar findings have been reported for FFA3. High fiber diet mediated protection against allergic airway disease and influenza virus is not observed in FFA3 knockout mice [85,87].

Metabolic studies have also highlighted the importance of the GM→SCFAs→FFA2 and FFA3 relationship. Reduced adiposity and PYY levels in FFA3 knockout mice are GM dependent, with the effect being lost in GF FFA3 knockout mice [63]. Similarly, GM-derived SCFAs mediate suppression of GIP secretion in FFA3 dependent manner, an effect lost in GF, antibiotic-treated mice (pseudo-GF), and FFA3 knockout mice [72]. In mice, a low-fiber diet or GF status during pregnancy increases the vulnerability of the offspring to obesity and insulin resistance later in life [92]. This effect could be rescued by propionate treatment or high-fiber feeding but not in absence of FFA3 or FFA2. Furthermore, FFA3 and FFA2 SCFA signaling was found to be responsible for normal embryonic development of neural, pancreatic β cell and intestine tissues. Collectively, these data suggest that the GM modulates metabolic and immune features affecting health via SCFA-FFA2 and SCFA-FFA3 axes.

## 4. FFA2 and FFA3 Mediating GM–Host Crosstalk in T1D

The identification of a role for FFA2 and FFA3 signaling in T1D is a budding area of research. Although there is still only sparse and indirect evidence, there is clinical interest in pursuing this area in the fight against T1D (discussed under “FFA2, FFA3, and T1D: Clinical Interests” (Section 5)). The main mechanisms linking GM to T1D include the GM-mediated influence on the development and homeostasis of the immune system and the effects of the GM upon influence on gut barrier integrity. As noted from GF and gnotobiotic mice studies, GM composition affects the development of gut-associated lymphoid tissue (GALT), the expansion and differentiation of specific T cell subsets (Foxp3^+^ Tregs and Th17 cells), and IgA-secreting B cells [93,94,95,96]. The gut barrier guards against the entry of pathogenic microbes and their components into the host circulation and tissues. Disruption of the gut barrier has been noted in both human T1D and T1D animal models [97,98,99]. This is manifested in humans by increased gut permeability [100] and serum levels of the gut barrier marker, zonulin [101]. In mice, it has been reported that there is activation of islet-specific diabetogenic T cells in the gut and their translocation along with gut bacteria to pancreatic lymph nodes [99,102].

**Figure 1:**
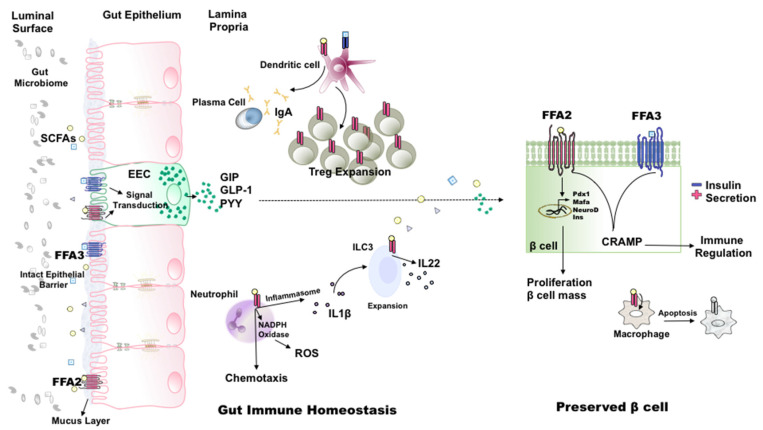
Role of FFA2 and FFA3 in gut immune homeostasis and β cell physiology in the context of T1D. The engagement of FFA2 and FFA3 on gut epithelial and enteroendocrine (EEC) cells by gut microbial metabolites, short-chain fatty acids (SCFAs), regulates epithelial barrier integrity and the secretion of various incretin hormones. FFA2 signaling on various gut immune cells promotes an anti-inflammatory and tolerogenic environment. Neutrophil FFA2 affects chemotaxis, the production of reactive oxygen species (ROS), and IL1β. FFA2 activation on innate lymphoid cell 3 (ILC3) directly and in conjugation with neutrophil-released IL1β promotes IL22 production. FFA2 also promotes ILC3 and regulatory T cell (Treg) expansion. Receptor activation on dendritic cells contributes to B cell (plasma cell) differentiation and IgA release. Altogether, SCFA receptor activity promotes an anti-inflammatory state that, in turn, suppresses the immune destruction of β cells. Factors produced in the gut, such as incretin hormones and SCFAs, and possibly immunosuppressive immune cells such as Tregs, travel to pancreatic β cells, influencing their physiology. In β cells, FFA2 stimulates insulin secretion and proliferation and is essential for the establishment and preservation of β cell mass. FFA3 activation reduces insulin secretion. Both receptors possibly stimulate the production of immunomodulatory cathelicidin-related antimicrobial peptide (CRAMP). FFA2 activation on infiltrating proinflammatory macrophages causes immune cell apoptosis.

Considering (1) the role FFA2 and FFA3 play in immune homeostasis and gut epithelial integrity, (2) dysbiotic gut microbiome of T1D, and (3) altered serum and fecal SCFAs in T1D, it can be argued that these receptors are likely to be important regulators of T1D immune responses (Figure 1). Along these lines, peripheral blood monocytes in T1D subjects show high FFA2 expression [103], and FFA3 expression has been correlated with inflammation and metabolic markers [104]. The first indication of the involvement of these receptors in T1D pathogenesis came from the work of Marino et al., [15], where the feeding of acetate yielding diets to NOD mice promoted immune tolerance and delayed progression to T1D by reducing autoreactive T cells, increasing Tregs, and improving gut barrier integrity. These effects were FFA2 dependent, as the anti-T1D potency of these diets was lost in NOD mice deficient in FFA2. Feeding a butyrate yielding diet, on the other hand, could confer partial protection from T1D to NOD FFA2-deficient mice. This indirectly suggested the involvement of butyrate-favoring receptor FFA3 and/or the butyrate-activated GPR109a or receptor-independent effects of butyrate. This study thus suggests that engagement of FFA2 and/or FFA3 by dietary SCFAs plays an important role in modulating inflammatory responses in T1D. A similar protective role of FFA2 was also observed in a streptozotocin-induced mouse model of T1D [103]. In diseased mice, treatment with specific FFA2 agonists attenuated islet inflammation by inducing apoptosis of infiltrating macrophages. Furthermore, these receptors, possibly through the Gα_i/o_-dependent pathway, were shown to promote a tolerogenic pancreatic immune environment by regulating islet production of immunoregulatory cathelicidin-related antimicrobial peptide (CRAMP) [105]. These effects, however, have not been replicated in humans. The use of FFA2 agonists in human studies is hampered due to differences in the signaling of mouse and human isoforms of the receptor. The more attractive option, oral administration of SCFAs, for example butyrate, in long-standing T1D subjects, unfortunately, has also not shown any benefits [106]. Whether or not oral SCFA administration prior to T1D onset can delay or lessen T1D pathology needs exploration. Moreover, FFA2 can also augment β cell function and protection, and both FFA2 and FFA3 can affect incretin secretion. However, it has not yet been explored whether these effects are pertinent to T1D.

## 5. FFA2, FFA3, and T1D: Clinical Interests

From the above discussion, a GM→SCFAs→FFA2 and FFA3→T1D link is apparent. This link has opened exciting avenues of research for identifying novel targets to treat and prevent T1D. The first question raised is if the modification of GM, which is achievable through the use of probiotics and prebiotics [107], impacts aspects of T1D disease. Probiotics are live microorganisms that, when administered in adequate amounts, confer a health benefit on the host, while prebiotics are substrates utilized by host microorganisms conferring a health benefit [108]. Several studies on spontaneous and pharmacologic rodent models of T1D have revealed that pro- and prebiotics favor a tolerogenic gut immune environment by promoting gut barrier integrity, stimulating the secretion of anti-inflammatory cytokines, and restricting the number of inflammatory T cell subsets besides increasing the abundance of beneficial gut bacteria [107]. Similar effects have been observed in human T1D trials [14,107]. Notably, early probiotic exposure in children genetically predisposed to T1D reduced the risk of the disease [109]. Interestingly, some probiotics such as *L. kefiranofaciens M* and *L. kefiri K* promoted GLP-1 secretion in a streptozotocin-induced T1D mouse model [110]. Presumably, FFA2 and FFA3 in EECs and gut immune cells are involved in some of these effects. This presumption may especially hold true for FFA2. For example, *Bifidobacterium animalis subsp. lactis* GCL2505 (GCL2505), a probiotic, increases acetate levels and engages FFA2 to exert beneficial metabolic effects in a diet-induced obesity mouse model [111]. Similarly, prebiotic fructo-oligosaccharide supplementation in rats increased the density of GLP-1 producing L cells coexpressing FFA2 [60]. Additionally, dietary supplementation with the microbially derived SCFAs, acetate and butyrate, ameliorated T1D β cell damage and immune dysfunction in an FFA2- and possibly FFA3-dependent manner [15]. Finally, FMT has appeared on the landscape of numerous anti-T1D interventions as another method to re-engineer the GM to positively impact T1D [30]. These different methods of modifying the GM for modulating the course of T1D seem potentially promising. However, we are still far from their actual clinical use. Human trials and animal studies have yielded variable outcomes ranging from no to even adverse effects [106,112]. Furthermore, the durability of these effects is unclear, while our understanding of gut microbial metabolite mediated cellular effects is incomplete.

Another new area of research in T1D therapeutics is the specific targeting of the receptors FFA2 and FFA3. Although this is seemingly straightforward compared to T1D GM modification, it comes with added complexities, as enumerated before [12]. Briefly, these are, biased G-protein coupling (FFA2) [113], species differences in GPCR signaling (FFA2) [37,114], multiple downstream effectors of the activated receptors yielding discrete physiological responses (FFA2 and FFA3) [36,37,44,66,115,116,117], lack of species-specific and G-protein-specific ligands (FFA2), lack of understanding of tissue-specific roles of these receptors, dependence of receptor expression on various factors such as diet [118], multiple factors such as diet, and multiple genetic polymorphisms in *FFA2* and *FFA3* without known associations with clinical/disease phenotypes [119]. Furthermore, how genetic predisposition to T1D affects SCFA receptor expression and activity in various tissues and conversely if polymorphisms in SCFA receptors confer risk to develop autoimmunity is not known. Despite these complexities, owing to the multiple ways these receptors can affect T1D, it is worthwhile to try closing these gaps in our knowledge and develop receptor-based T1D interventions. One step forward in this direction is to develop preclinical mouse models, such as NOD with double receptor knockout and the tissue-specific knockout of FFA2 and FFA3, for the precise delineation of receptor-mediated effects. Another approach is to generate humanized mouse models expressing human isoforms of the receptors globally and in tissue-specific manners through knock-in and chemogenetic approaches that will provide excellent ways to monitor effects of human receptor signaling in in vivo settings and identify unique signaling responses to new ligands. The generation of such models can be used to demonstrate the functional implications of GM changes occurring in T1D.

## 6. Conclusions

Mounting evidence indicates an effect of the GM upon T1D pathology mediated through the modulation of gut and pancreatic immune environments. With possible roles of the SCFA-activated GPCRs, FFA2 and FFA3, in mediating these effects, a highly relevant GM→SCFAs→FFA2/FFA3→T1D link is apparent. Although a mechanistic understanding of the interrelationships of different entities in this link is still not complete, novel therapeutic interventions against T1D based on this link are likely to emerge.

## Figures and Tables

**Table 1:** Affinity (EC50 in μM) of SCFAs at their cognate receptors.

SCFA	FFA2	FFA3
Acetate	35–431	>1000
Propionate	14–290	6–127
Butyrate	28–371	42–158
Pentanoate	>1000	42–152
Hexanoate	-	102–134

All values are for human receptors [35,36,37].
